# 162. Assessment of Guideline-Concordant Testing for *Legionella pneumophila* and *Streptococcus pneumoniae* in Community-Acquired Pneumonia

**DOI:** 10.1093/ofid/ofac492.240

**Published:** 2022-12-15

**Authors:** Waleed Malik, Ann Fisher, Brian Kotansky, Louise Dembry, Rupak Datta

**Affiliations:** NYU School of Medicine, New York, New York; Yale University, New Haven, Connecticut; VA Connecticut Healthcare System, West Haven, Connecticut; Yale University, New Haven, Connecticut; Yale University, New Haven, Connecticut

## Abstract

**Background:**

Urinary antigen testing for *Streptococcus pneumoniae* and *Legionella pneumophila* is only recommended in patients with severe pneumonia according to the 2019 guidelines from the American Thoracic Society (ATS) and Infectious Diseases Society of America (IDSA). Unnecessary testing for these organisms may increase antibiotic utilization. This quality improvement project evaluated whether patients who received a *Streptococcus pneumoniae* or *Legionella pneumophila* urine antigen met criteria for testing.

**Methods:**

We conducted a cohort study of patients who received a urinary antigen test for *Streptococcus pneumoniae* or *Legionella pneumophila* within 3 days of admission to the Veterans Affairs Connecticut Healthcare System between September 1, 2018 and September 1, 2021. We determined whether patients had clinically defined pneumonia per National Healthcare Safety Network criteria. Among patients that met criteria for clinically defined pneumonia, the subset with severe pneumonia according to the ATS/IDSA guidelines was identified. We used the ATS/IDSA guidelines to determine whether patients received guideline-concordant choice of therapy.

**Results:**

We identified 352 patients who received a urinary antigen test within 3 days of admission. Mean age was 72.6 years, 97% (n=278) were male sex, and 86% (n=301) were White race. Common comorbidities included heart disease (76%, n=268), lung disease (58%, n=203), and diabetes (30%, n=106). Overall, 36% (n=127) of patients met criteria for clinically defined pneumonia, and 7% (n=25) met criteria for severe pneumonia. 9 patients had a positive *Streptococcus pneumoniae* antigen test and 1 patient had a positive test for *Legionella*. Among patients with clinically defined pneumonia, 74% (n=75) received guideline-concordant choice of therapy and 74% (n=75) received an antibiotic duration >5 days

Diagnostic and Therapeutic Characteristics of Veterans According to Presence of Pneumonia

**Figure:**
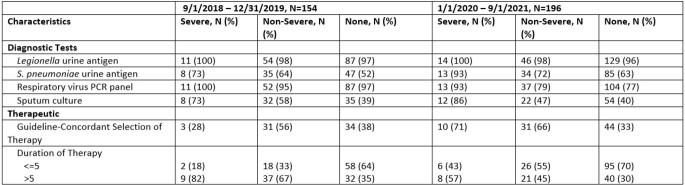

**Conclusion:**

Only 7% of patients who underwent urine antigen testing for *Streptococcus pneumoniae* or *Legionella pneumophila* met criteria for testing per ATS/IDSA guidelines, and length of therapy was prolonged in the vast majority of patients. These data support the need for a clinical decision-making tool to reduce unnecessary testing and treatment for pneumonia at our institution.

**Disclosures:**

**All Authors**: No reported disclosures.

